# Cardiac Damage After SARS-CoV2 Infection

**DOI:** 10.7759/cureus.60641

**Published:** 2024-05-19

**Authors:** Ben Bohlen, Damian Franzen

**Affiliations:** 1 Cardiology, Medizinisches Versorgungszentrum (MVZ) Franzen Institut, Cologne, DEU

**Keywords:** covid-19, viral infection, long covid syndrome, covid-19 long term outcomes, cardiac risk factors and prevention, corona virus, electrocardiography (ecg), 2-dimensional echocardiography

## Abstract

COVID-19 is a viral disease that can manifest acutely in the respiratory tract and other organs. In this study, we aimed to investigate potential long-term damage to the heart from COVID-19. For this study, we divided 97 consecutive unselected COVID-19 patients aged 18-80 years at a cardiology practice in Cologne, Germany, into two groups based on the severity of their infection. We performed a resting ECG and a resting transthoracic echocardiography three and six months after SARS-CoV2 infection. The key discriminator determining disease severity was bed confinement or hospital admission. Group 1 included patients with less severe COVID-19, whereas group 2 contained more severe cases. Heart rate as the primary ECG endpoint was lower by a statistically significant amount for the entire study population (p=0.024), subdivided by gender (p_women_ <0.001, p_men_ <0.001) and in group 1 p =0.003 compared to three months. QTc time and repolarization disturbances as primary ECG endpoints and the echocardiographic primary endpoints, left ventricular ejection fraction, and left ventricular end-diastolic diameter (LVEDD), showed no relevant difference between the subgroups at three and six months or between the measurements taken at each point. In contrast, LVEDD normalized to body surface area was statistically significantly lower at six months in women in group 1 compared to group 2 (p=0.048) and in the overall study population at six months compared with the data after three months (p=0.034). E/E' was statistically lower at six months than at three months in the whole population (p=0.004) and in women (p=0.031). All measured echocardiographic and electrocardiographic mean values were within the normal range in all groups and follow-up controls. Overall, the prospective study conducted showed no significant evidence of long-term cardiac damage from COVID-19 disease, as evidenced by electrocardiographic and echocardiographic examinations at three and six months after infection.

## Introduction

Coronavirus disease 2019 (COVID-19), the disease caused by the severe acute respiratory syndrome coronavirus type 2 (SARS-CoV2), was first identified in China in 2019 [1], and it eventually developed into a pandemic [2]. Several months after the disease's initial description, researchers and clinicians noticed multi-organ affection in patients and characterized potential relevant cardiac changes [3,4]. The cardiac manifestations during the acute course of the disease were characterized rapidly [3,5-8], but little is known about the possible long-term cardiac damage caused by COVID-19. Thus, serial cardiac examinations following a SARS-CoV2 infection have the potential to both clarify further cardiac impacts and detect potential late cardiac damage not seen at earlier stages. The goal of this study was to identify electrocardiographic and echocardiographic signs of cardiac involvement at three and six months after the acute phase of infection. The significant number of patients who survived COVID-19 might be a good base to further study cardiac damage in a noninvasive, standardized, and low-cost manner.

## Materials and methods

Study design

We recruited male and female nonselected study participants from a cardiac outpatient setting, MVZ Franzen Institut, in Cologne, Germany, between December 2020 and December 2021. We conducted follow-ups at three and six months after acute SARS-CoV2 infection using a 12-lead ECG and echocardiographic examination at rest. The study was designed as a prospective, nonblinded, nonrandomized, mono-centric observational study. The study was approved by the ethical committee of the medical association North-Rhine (AEKNO) under the registration number 2012178 and is registered in the German Clinical Trials Register under the number DRKS00023831. We obtained informed consent from all individual participants included in this study.

We classified two groups of participants according to the severity of their symptoms during the acute phase of infection, as listed in Table 1. Group 1 contained participants who were asymptomatic or oligosymptomatic, with no clear evidence of pneumonia or severe COVID-19. These participants were not bedridden for more than a day and did not have an altered mental state. The second group contained participants who were bedridden, had an altered mental state, or evidence of COVID-19 pneumonia, and/or were treated in an inpatient hospital setting. There was no differentiation between participants being treated in and outside an intensive care unit (ICU). We planned to include at least 30 participants in each group to achieve a sufficient statistical power of 75% using the student's t-test with a significance level of p<0.05 and a medium effect using Cohen's d.

**Table 1:** Classification of groups Classification of study participants in group 1 and 2 according to their symptoms during the SARS-CoV2 infection. PCR - polymerase chain reaction; SARS-CoV2 - severe acute respiratory syndrome corona virus type 2; ICU - intensive-care unit; ARDS - acute respiratory distress syndrome

Classification criteria	Group 1	Group 2
PCR proof of SARS-CoV2	Yes	Yes
Symptoms	Yes/No	Yes
Bedridden	No	Yes
Out- or inpatient setting	Outpatient	Out- and inpatients
Altered mental status	No	Yes
ICU or ARDS	No	Yes

Inclusion and exclusion criteria

Inclusion criteria were defined as being between 18 and 80 years, having signed informed consent, and a PCR-confirmed first infection with SARS-CoV2. A history of myocarditis or heart transplant, as well as the presence of an implanted cardiac pacemaker, a terminal renal insufficiency, and dementia, were defined as the exclusion criteria. These exclusion criteria were chosen because of possible misinterpretations of electrocardiographic [9] and echocardiographic [10,11] alterations. Participation was voluntary.

Basic investigations

We measured age, blood pressure [12], body weight, and height for every participant taking part in this study and used these demographics in the statistical analysis.

ECG

We performed a 12-channel ECG for each participant (Custo Cardio 200, Custo Med, Ottobrun, Germany) and recorded them under resting conditions at three and six months after infection. We used a writing speed of 50 mm/ms, a filter of 0.05-150 Hz, and a calibration of 1 mV/cm. We mounted the ECG electrodes using WHO standards and recorded the ECG online using Medys 10 software, version 62_50, and analyzed them offline. We chose heart rate, p-wave length, PR interval, QRS interval, and QT interval (absolute, Bazett-corrected QTc, and relative) as primary and secondary endpoints [13,14,15]. We also recorded and analyzed the rhythm, heart axis, bundle block, and changes in depolarization (changes in the ST-segment, T-wave inversions) [13-18]. Table 2 presents the normal values [9].

**Table 2:** Classification of normal values in the 12-lead ECG n.s. - no specification; mV - millivolt; ms - milliseconds; bpm - beats per minute

ECG-parameter	Normal value	Amplitude	Pathological
Heart frequency (bpm)	50-100	n.s.	<50 and >100
P-wave	50-100 ms	<0.25 mV	>100 ms; >0.25 mV
PR-IntervalHF > 60 bpm	120-200 ms	n.s.	>200 ms
PR-Interval HF < 60 bpm	120-220 ms	n.s.	>220
Q-wave	<40 ms	<1/4 R	>40 ms and >0.3 mV
QRS-interval	60-120 ms	n.s.	>120 ms
T-wave	n.s.	1/6 - 2/3 R	see ST-segement
QTc(B)-interval (men)	Man 320-460 ms	n.s.	>460 ms and <320 ms
QTc(B)-interval (woman)	Women 320 - 450 ms	n.s.	> 450 ms and < 320 ms

Transthoracic echocardiography

We used transthoracic echocardiography as a noninvasive, reproducible, and unsophisticated procedure to visualize anatomical and physiological structures and measure parameters in real time. We used a GE Healthcare Vivid T8 device with a 3.5 GHz sectorprobe (GE Healthcare, Norway) and recorded the two-dimensional images using an ECG-triggered loop, with the participant lying in the left lateral position. We acquired the images in the parasternal short and long axis, as well as apical views. We used B-images and M-mode, as well as cw- and pw-Doppler, to evaluate cardiac function. We calculated the left ventricular ejection fraction (LV-EF) using the "auto-EF" feature in the two- and four-chamber views [10]. We presented the two- and four-chamber views to the machine, which differentiated the myocardial borders from the blood-loaded cavum of the left ventricle [10]. The algorithm captured the border from the medial annulus of the mitral valve along the inferoseptal border to the apex and along the anterolateral myocardial wall to the lateral annulus of the mitral valve, as seen in the four-chamber view [10]. In the two-chamber view, the machine captured the border from the inferior annulus of the mitral valve along the inferior wall of the left ventricle to the apex and along the anterior wall of the left ventricle to the anterior annulus of the mitral valve [10]. We then confirmed the tracing, and the machine calculated the area of the left ventricle in diastole and systole.

**Figure 1:**
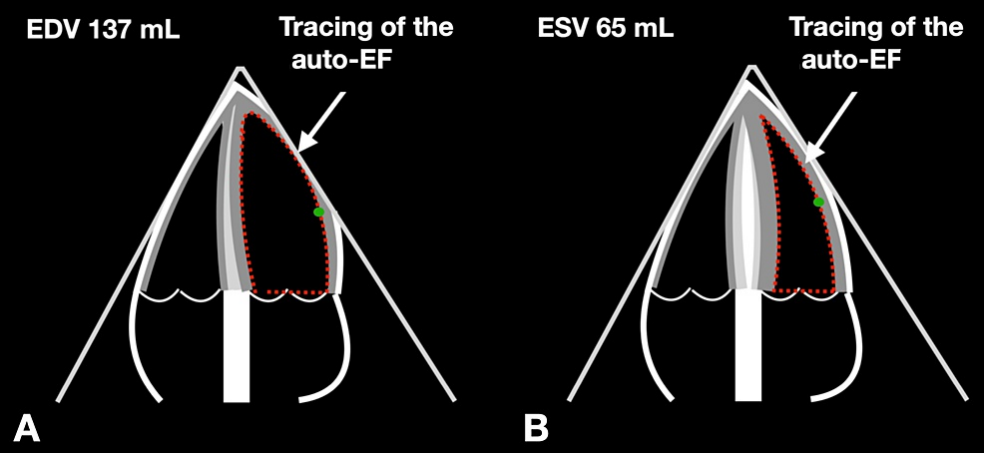
Auto-EF This figure shows schematics of GE's auto-EF, which delineates the blood-filled cavum of the left ventricle from the myocardial border in diastole and systole. Using the end-diastolic and end-systolic volumes, GE can aid with calculating the left ventricular ejection fraction online.

Using the planimetry method, we estimated the end-diastolic and end-systolic volume and generated the ejection fraction (EF, in %) and the stroke volume (SV, in ml), as shown in Figure 1. We only manually changed the automatically-generated tracing of the left ventricle in the case of coarse failure of the machine's algorithm. We measured the geometry of the left ventricle using M-mode in the parasternal long axis with the LV-study. The M-mode was pointed vertically at the interventricular septum, from which position we recorded several heartbeats [11]. We measured the left ventricular end-diastolic diameter (LVEDD) shortly after the QRS complex at the moment when the LV was maximally dilated [11]. We also measured the interventricular septal thickness in diastole (IVSd) shortly after the QRS complex [11]. We analyzed LV-EF, the LVEDD, and the LVEDD standardized to the participant body surface area (BSA - LVEDD/BSA) [11]. We calculated the diastolic function of the left ventricle using functional and morphological echocardiographic parameters [11]. We estimated the velocity of the myocardial contraction using the pw-Doppler in tissue Doppler imaging at the medial annulus of the mitral valve (E') [11], and we measured the E/A ratio in the pw-Doppler signal across the mitral valve [11].

We investigated pericardial effusion, which can best be seen on B-mode as an echo-free space, in every view [11]. Table 3 lists the reference values used [11].

**Table 3:** Classification of normal values in the TTE LVEDD - left ventricular end-diastolic diameter; BSA - body surface area; LV-EF - left ventricular ejection fraction; LA - left atrium; IVSd - interventricular septum in diastole; mm - millimeter; ms - millisecond; m2 - square meter; n.s. - no specification

Echocardiographic parameter	Normal values		Pathological					
	Men	Women	Mild		Moderate		Severe	
			Men	Women	Men	Women	Men	Women
LV-EF (%)	52-72	54-74	41-51	41-53	30-40	30-40	<30	<30
LVEDD (mm)	42-58	38-52	59-63	53-56	64-68	57-61	>69	>61
LVEDD/BSA (mm/m^2^ KOF)	22-30	23-31	31-33	32-34	34-36	35-37	>36	>37
E-wave	n.s.							
E' septal	> 7 cm/s		< 7 cm/s					
E' lateral	> 10 cm/s		< 10 cm/s					
E/E'	< 14		> 14					

Statistical analysis

We carried out the statistical analysis with SPSS version 28.0.1.0 (IBM Inc, Armonk, New York). We presented numeric data as mean values, with one standard deviation. Normally, we evaluated the distributed values using the student's t-test. We evacuated nonnormally distributed parameters using the Mann-Whitney U Test, and we determined the classification of normally and nonnormally distributed values with the Shapiro-Wilk test and by interpreting the normal curve of distribution. We compared the values across the two presentations, across the two groups, and with reference values, if feasible. We accepted a statistical significance with a p-value of less than 0.05. We presented the categorical variables in percentage form, defining heart frequency (HF, in bpm) and repolarization as the primary endpoints in the electrocardiographic study. We defined pathological repolarization as a non-physiological or pathological change in the ST-segment, T-wave inversion, and pathological QTc interval. We chose changes in the heart axis over the two presentations and pathological depolarization (prolonged PR interval) as secondary endpoints in the electrocardiographic study. We chose the LVEDD, measured with M-mode in the parasternal long axis, and LV-EF, calculated with GE's "auto-EF", as primary endpoints in the echocardiographic study. We defined the presence of pericardial effusion and signs of diastolic dysfunction as secondary endpoints in the echocardiographic study.

We scaled the categoric variables on a binary. The presence of a pathological repolarization in the ECG, bundle blocks, and the presence of a pericardial effusion are categorical variables. Numeric variables in the ECG are the heart rate and length of PR, QRS, and QT intervals. Numeric variables in the echocardiographic study are the LVEDD, LV-EF, E, E', and E/E'.

## Results

Study population

We included 97 participants in the study, of whom 40.2% (n=39) had a mild or oligosymptomatic SARS-CoV2 infection (group 1), and 59.8% (n=58) had a severe or symptomatic infection (group 2). Overall, 57.7% (n=56) were male, and the average age was 47.1 years (18-76 years ± 15.5 years, men 50.5 years, women 42.5 years). Age was not associated with infection severity and gender. Weight and BMI also did not have significant relationships to infection severity and gender, but there was a clear trend toward a higher weight and BMI in group 2. Table 4 shows patient characteristics.

**Table 4:** Descriptive statistics regarding symptoms and pre-existing conditions Patient characteristics in group 1 and 2 based on their symptoms and criteria Data given as patients (n) and percentage (%) *during the acute infection

Patient characteristics	Total	Group 1	Group 2
Mild course	39 (40.2 %)	39 (100 %)	0 (0 %)
Severe course	58 (59.8 %)	0 (0 %)	58 (100 %)
Gender (men)	56 (57.7 %)	27 (69.2 %)	29 (50 %)
Fever	58 (59.8 %)	16 (41 %)	42 (62.4 %)
No fever	39 (40.2 %)	23 (59 %)	16 (27.6 %)
Bedridden *	58 (59.8 %)	0 (0 %)	58 (100 %)
Cough *	41 (42.3 %)	7 (17.9 %)	34 (58.6 %)
Dyspnoea *	42 (43.3 %)	9 (23.1 %)	33 (56.9 %)
Altered mental status *	16 (16.5 %)	0 (0 %)	16 (27.6 %)
Inpatient treatment *	15 (15.5 %)	0 (0 %)	15 (25.9 %)
Intensive care treatment *	9 (9.3 %)	0 (0 %)	9 (15.5 %)
Cardiac symptoms *	36 (37.1 %)	7 (17.9 %)	29 (50 %)
Persisting symptoms after 3 months	35 (36.1 %)	5 (12.8 %)	30 (51.7 %)
Active smoker	5 (5.2 %)	1 (2.6 %)	4 (6.9%)
Non-smoker	78 (80.4 %)	33 (84.6 %)	45 (77.6 %)
Former smoker	7.1 (7.2 %)	3 (7.7 %)	4 (6.9 %)
Arterial hypertension	16 (16.5 %)	5 (12.8 %)	11 (19 %)
Diabetes mellitus type II	3 (3.1 %)	1 (2.6 %)	2 (3.4 %)
Chronic coronary syndrome	1 (1 %)	0 (0 %)	1 (1.7 %)

Overall, 66 participants followed up for both the three-month and the six-month measurements. In total, the mean systolic and diastolic arterial blood pressure of all patients was statistically significantly lower after six months compared with the measurement after three months (p_sys_=0.009 and p_dia_=0.002). Diastolic blood pressure was overall lower in women compared to men (p=0.044), but after six months, no relevant difference was visible.

Descriptive analysis of the ECG examination

We defined pathological depolarization patterns, including ST-segment and T-wave alterations, as primary endpoints. After three months, we saw T-wave inversions (TWI) in 27.1% of the participants, whereas after six months, we saw TWI in only 22.7%. Nonspecific elevation or depression of the ST segment that did not fulfill the criteria of ischemia did not show a higher incidence in either of the groups and did not change over time.

We defined the change of the heart axis as a secondary endpoint. After three months, we saw 54.4% with an indifferent type, 23.9% with a left axis deviation, and 14.1% with a vertical type. Only 5.4% showed an extreme axis deviation. We did not observe a right-axis deviation in this study. Over the study period, only one participant developed an extreme (left) axis deviation with features of a left anterior bundle branch block (LABBB).

We observed sinus rhythm in 98.9% of subjects. One group 2 participant, who had been treated in the ICU, showed a ventricular bigeminus after three months. At the second presentation, he presented with sinus rhythm. After three months, 6.7% of the participants were tachycardic, and 3.9% were bradycardic. After six months, only one participant had tachycardia, and one other patient was bradycardic (each 1.3%). Paroxysmal supraventricular tachycardias and malignant ventricular tachycardias were not registered at either test time.

After three months, 68.5% of the subjects showed no specific bundle block. 20.6% showed an incomplete right bundle branch block (iRBBB), and 2.2% showed a complete right bundle branch block (cRBBB). The iRBBB was more prevalent in group 2. Additionally, 2.2% showed a first-degree AV block and 4.4% a LABBB. We did not document a complete left bundle branch block in this study. LABBB and cRBBB did not show a higher prevalence regarding the groups. We saw three changes over time. One participant from group 1 developed a second-degree AV block type Mobitz with the loss of a single QRS complex in the resting ECG. The participant was asymptomatic, and the block was not recorded. The second participant developed a first-degree AV block after six months. The third case showed no block after six months but did show an iRBBB after three months.

Statistical analysis of the ECG examination

All primary and secondary endpoints except the QRS complex, QTc(B) interval, and QT-time in percentage showed a normal distribution after three months (after six months for the PR interval), and we investigated them using a student's t-test.

In the total population, heart frequency (HF) as a primary endpoint was lower after six months than after three months (p=0.024, Table 5). There was no difference after six months between groups 1 and 2 or between genders. Nevertheless, we saw a statistically significant difference in HF after three months between groups 1 and 2, with a lower HF in group 2 (76.9 bpm in group 1 versus 72.8 bpm in group 2, p=0.003, Table 5).

**Table 5:** Statistical analysis of primary and secondary ECG-endpoints Statistical analysis of primary and secondary ECG-endpoints compared over time after three and six months in the total study population and subdivided regarding subgroups. HF - heart frequency; N - number; p - significance; ms - milliseconds

Parameter after 3 and 6 months	Total		Group 1		Group 2	
	N	p	N	p	N	p
HF at rest	66	0.024	27	0.003	39	0.479
PR interval	69	0.480	28	0.283	41	0.271
QTc(B) interval	69	0.428	28	0.177	41	0.301
QT time in ms	69	0.265	28	0.026	41	0.187
QT time in %	68	0.500	27	0.260	41	0.308

The QTc(B) interval as part of the primary endpoint repolarization did not show a difference after three and six months between groups 1 and 2 or regarding gender.

The PR interval as a secondary endpoint was significantly shorter in group 2 than in group 1 after three (p=0.016) and six (p=0.015) months. This was consistent with the PR interval in women being shorter in group 2 after three months compared to women in group 1 (p=0.037). For men, no significant difference was observed after three months.

A secondary finding was a shorter QRS complex after six months in participants who had been treated in the ICU during their SARS-CoV2 infection compared to their QRS complex after three months (p=0.019). Beyond that, the QRS did not show a significant difference.

We tested all primary and secondary endpoints against reference values from a study performed by Mendzelevski et al. [19] with 79.743 investigated probands. That study further subdivided each parameter into age cohorts, which we did not do; instead, we used the mean values without adjustments for age. 

In the total study population, we found HF was statistically significantly higher after three and six months compared to the reference (mean) values (p_after three months_<0.001 and p_after six months_=0.016). This finding was consistent after three months and subgroup-independent (p_group1_<0.001 and p_group2_=0.009). After six months, only group 2 showed a higher HF compared to the reference value (p=0.027).

The QTc(B) time was significantly higher in the total study population and in the subgroup analysis after three and six months compared with the reference values.

The PR interval was statistically significantly higher in group 2 after three months (p=0.009) and in group 1 after six months (p=0.020), as well as in female probands after three months (p=0.027).

Statistical analysis of the echocardiographic examination

All parameters except E' and E/E' after three months, as well as E' after six months, were normally distributed, and we investigated them using a student's t-test.

LV-EF and LVEDD did not show a significant difference between groups 1 and 2 after three and six months. There was a significantly lower LVEDD/BSA after six months in group 1 compared to group 2 (p=0.034). E' and E/E' as secondary endpoints did not show a significant difference between groups 1 and 2 after three and six months.

We further analyzed the primary and secondary endpoints by gender. The LV-EF showed no significant difference between groups in men and women. The LVEDD at three months showed a significant difference in women; this value was significantly lower in female participants in group 1 than in the female population in group 2 (p=0.048). In contrast, LVEDD normalized to BSA in women failed to reach statistical significance in women (p=0.067). After six months, we found no statistically significant difference in LVEDD or LVEDD/BSA among female participants in group 1. There was no difference between male group members in either study. E' was statistically significantly higher in women in group 1 compared to women in group 2 at three months (p=0.031). E' at six months and E/E' at both presentations in women showed no statistically significant difference. No statistical effect was found in men.

We also performed comparisons between the first and the second presentations (Table 6). The LV-EF, as well as E', did not show a significant difference between both presentations. The primary endpoints LVEDD and LVEDD/BSA and one of the secondary endpoints E/E' were statistically higher after three months in the total population (p_^LVEDD^_=0.002, p_LVEDD/BSA_=0.002, and p_E/E'_=0.004) compared to the second presentation after six months. This effect can also be observed in the subgroup analysis.

**Table 6:** Statistical comparison of TTE-parameters after three and six months Longitudinal comparison of left-ventricular ejection fraction (LV-EF), parameters of diastolic function (E', E/E'), left ventricular end-diastolic diameter (LVEDD) und LVEDD normalized to body surface area (LVEDD/BSA) after three and six months and divided into subgroups. N - number; p - statistical significance

Parameters	Total		Group 1		Group 2	
	N	p	N	p	N	p
LV-EF after 3 and 6 months	66	0.1	27	0.223	39	0.156
E’ after 3 and 6 months	48	0.306	18	0. 371	30	0.353
E/E’ after 3 and 6 months	48	0.004	18	0.025	30	0.033
LVEDD after 3 and 6 months	62	0.002	23	0.015	39	0.029
LVEDD/BSA after 3 and 6 months	62	0.002	23	0.011	39	0.028

Furthermore, we compared the primary and secondary endpoints with reference values defined by the European Association of Cardiovascular Imaging (EACVI) and the American Heart Association (AHA) [11]. In women, we defined a normal LV-EF as 64% (±5%, one standard deviation) [11]. The averaged LV-EF in women was statistically significantly lower after three and six months compared to the reference value with one standard deviation (both p<0.001). In men, a normal LV-EF is defined as 62% (±5%, one standard deviation) [11]. The mean LV-EF in men after three and six months was also statistically significantly lower (both p<0.001). Using the reference value with two standard deviations, we found mirror images. For women, an LV-EF ≥ 55% is defined as a normal EF, whereas for men, an LV-EF of >52% is considered normal [11]. In all study participants, LV-EF was statistically significantly higher than the lower limit of the normal LV-EF defined in the reference values (p<0.001). This effect was also found in the subgroup analysis between group 1 and group 2. LVEDD in women in the total population (p_three months_=0.001 and p_six months_=0.009) and in group 2 (p_three months_<0.001 and p_six months_=0.008) was statistically significantly higher than the reference value at three and six months. In group 1, there was no statistical effect in LVEDD compared to the reference value at either presentation (p=0.222 and p=0.434). Male participants in the total study population had a statistically significantly higher LVEDD compared to the reference value at three months (p=0.003). After six months, no statistical effect could be demonstrated (p=0.276). In the subgroup analysis, we found a higher LVEDD after six months in group 2 (p=0.011). LVEDD/BSA was not statistically different in women after three and six months or in men after three months. After six months, LVEDD/BSA was lower in men in the total population (p=0.004). In the subgroup analysis, LVEDD/BSA was lower for men and women in group 1 after six months (p=0.01 and p=0.042, respectively) compared to the reference values. The reference values for E and E/E' were age-dependent, so, given our relatively small study population, we did not compare study E and E/E' data with reference values.

A pericardial effusion was also defined as a secondary endpoint. Accompanied by an elevated LVEDD, LVEDD/BSA, or wall motion abnormalities, pericardial effusion is suggestive of myocarditis. In this study, we detected no pericardial effusion by transthoracic echocardiography after three and six months.

## Discussion

Our study investigated long-term cardiac effects after SARS-CoV2 infection in an unselected participant collective of a cardiac outpatient office in Cologne, Germany. The participants were examined after three and six months via electrocardiogram and echocardiogram to detect potential damage to the heart.

Systolic and diastolic blood pressure were statistically significantly lower after six months (p=0.009) in the total study population. At both presentations, we recorded participants' blood pressure as being within normal reference values for both groups 1 and 2 (<140 mmHg in systole and <90 mmHg in diastole). Systolic blood pressure did not show a statistical effect in subgroup analysis, whereas diastolic blood pressure was significantly lower in women after three months compared to men (p=0.044). Whether blood pressure is affected by SARS-CoV2 infection remains unclear [12].

ECG

Almost all participants presented with a sinus rhythm, some with sinus tachycardia that resolved over time. Supraventricular or ventricular rhythm disorders could not be documented at either presentation. Only one participant in group 2 developed a relevant sinoatrial block. Supraventricular tachycardias and bradycardias are well-described in the acute phase of the infection [13-15].

Nearly 70% of participants had no heart block at the first presentation. The most common heart block in this study was an incomplete right bundle branch block (occurring in 21% of subjects, compared to a prevalence of 4.5-9% in the European population overall) [16]. We documented a decline in iRBBB prevalence at the second presentation, suggesting a normalization of the right heart stress and hemodynamics. Bussink et al. showed an iRBBB without underlying structural heart disease was not associated with increased all-cause mortality [17]. In contrast, Nielsen et al. found an iRBBB was associated with the manifestation of paroxysmal atrial fibrillation [18]. It remains a question for further studies whether an iRBBB, which seems to be a frequent heart block after a SARS-CoV2 infection, can be associated with this rhythm disorder. This heart block appears to be relevant for follow-up care, as a persisting iRBBB or the development of a complete right bundle branch block (cRBBB) suggests persistent cardiovascular damage. Normalization of the iRBBB, in contrast, suggests a prognostic-favorable development.

Missing preliminary ECG recordings are a limitation in our study. Due to the assumptive prevalence of the iRBBB, as mentioned earlier, we can assume the majority of subjects' iRBBBs were nonexistent prior to SARS-CoV2 infection. We did not see an increase in the prevalence of cRBBB. We saw an atrioventricular block in two participants in group 1 at the first presentation. At the second presentation, we saw a normalization of the PR interval in one participant, suggesting the AVB could be due to COVID-19. A different participant in group 2 developed an AVB at the second presentation. Multiple studies demonstrated a dynamic AVB during the acute phase of COVID-19 [13-15]. 

Primary endpoints: repolarization

The QT interval (absolute and corrected after Bazett's formula) did not show a statistical effect over either presentation or in subgroup and gender analysis. This means there was no sustained prolongation of the QTc(B) interval after three and six months.

In more than 25% of participants, we documented singular or multiple T-wave inversions (TWI) that declined over time, suggesting normalization of myocardial repolarization.

Many participants complained about palpitations as a consequence of extrasystoles, sinus tachycardia, or postural orthostatic tachycardia syndrome after COVID-19. We saw a statistically relevant difference in heart frequency at rest in the total population and when separated by gender over both presentations. After six months, HF was statistically significantly lower (p_total_=0.024 and p_women/men_<0.001). Furthermore, we saw a statistically significantly lower HF after six months compared to the HF after three months in group 1 (p=0.003). There was no statistical effect comparing women and men over both presentations. It is worth mentioning the mean HF was always within normal HF ranges at rest. Nevertheless, it shows a clear trend of changes in HF after COVID-19. Szekely and Lichter also showed that, during the acute phase of COVID-19, HF is positively associated with the severity of SARS-CoV2 infection [20]. Our findings suggest an accelerated heart rhythm up to a sinus tachycardia can develop independently of infection severity, but this can resolve over time. The fact that we also saw a clear decrease in participants with sinus tachycardia strengthens this thesis. 

Secondary endpoints

The majority of participants showed a physiological heart axis at the first presentation. After six months, we saw a numeric increase in LABBB, with an extreme left axis. We did not observe a right or extreme right heart axis in this study. At the first presentation, we saw a statistically longer PR interval in group 1 compared to group 2 (p=0.016), which was persistent at the second presentation (p=0.015). The mean PR interval was within normal ranges in both groups. We expected the opposite result, as it is well-documented that acute SARS-CoV2 infection with cardiac affection can lead to a dynamic atrioventricular block [13-15].

Transthoracic echocardiography

Primary endpoints

LV-EF describes the pumping capacity of the left ventricle and is best measured by planimetry [11]. Both the absolute value and the chronological sequence of the LV-EF play a key role in indicating myocarditis. In this study, we chose the normal EF reference values of ≥55% for women and ≥52% for men [11]. The mean LV-EF in our study population did not show a statistical effect in either the presentation or the subgroup analysis. There was no increase or decrease in LV-EF over time or between subgroups, suggesting there is no relevant myocardial affection. GE's "auto-EF" proved to be a reliable and practical method of examination to quantify LV-EF in two planes. Abazid et al. showed there is no statistical difference between "auto-EF" and visual estimation [21]. We conclude there was no reduction in LV-EF over time after SARS-CoV2 infection, suggesting no indication of persisting or relevant myocarditis; however, Boehmer et al. suggested COVID-19 is associated with a 16-fold increase in the risk of myocarditis compared to a healthy population [22]. The course of LV-EF after COVID-19 was parallel to that described by Szekely and Lichter, as well as by Fayol and Antoine [20,23]. Neither study found a decrease in LV-EF independent of the severity of COVID-19 [20,23].

LVEDD is gender-dependent and should be normalized to body surface area (LVEDD/BSA) [11]. In this study, we could not find a difference between LVEDD in groups 1 and 2 at either presentation. LVEDD was lower in women after three months in group 1 compared to group 2 (p=0.048). After six months, we found a statistically significant difference in LVEDD/BSA between groups, with a lower LVEDD/BSA in group 1 than in group 2 (p=0.034). Comparing LVEDD and LVEDD/BSA in the chronological sequence, we found lower values after six months in groups 1 and 2. It is again worth mentioning that all values were within normal ranges. Nevertheless, these results suggest a trend toward a smaller diameter of the left ventricle. We conclude there was no clinically relevant difference in LVEDD and LVEDD/BSA between group 1 and 2 participants, although statistically significant differences were observed. Rathore et al. found that SARS-CoV2-associated myocarditis was associated with LV dilatation in just 8% of patients [23,24].

Secondary endpoints

The precise determination of the diastolic function is complex and time-consuming. In our study, we focused on E/A, E', and E/E', given the inability to define diastolic function following the HFA-PEFF-Score [25] and current guidelines [11]. Groups 1 and 2 did not differ at either presentation regarding E' and E/E'. In the total study population and subdivided subgroups, E/E' was statistically significantly lower after six months (p=0.004), but again, both mean values were within physiological ranges. In conclusion, we did not find indications of persistent diastolic dysfunction after COVID-19. Only one participant fulfilled all the criteria of diastolic dysfunction after three months. At the second presentation, this participant had a normalized E/E' that no longer fulfilled the criteria. Szekely and Lichter showed diastolic function is more often affected than systolic LV function [20]. A further key point of this investigation was the finding that there was no difference in diastolic function between patients with moderate and severe courses of COVID-19 [20].

A pericardial effusion can indicate a peri- or myocarditis affection from SARS-CoV2 infection. In this study, no pericardial effusion was present at either presentation. Taken together, the normal LV-EF and LVEDD or LVEDD/BSA, as well as the missing pericardial effusion, strengthen the interpretation that there was no relevant pericardial and myocardial affection [7].

Limitations

One limitation of this study is its sample size. The relatively low number of patients included is due to the study's mono-centric design and relatively short inclusion period, as well as the lack of active recruitment via general physicians or other sources. The source of subjects, a cardiology clinic, likely also introduced some selection bias because patients were referred to the clinic due to their symptoms, and people not suffering from cardiological symptoms would not have received a referral. However, this does lead to the thesis that the majority of patients surviving COVID-19 do not suffer from relevant cardiac effects, although a much larger sample size would be needed to demonstrate that thesis statistically. 

Finally, a more detailed echocardiographic examination of subjects, including a full work-up of diastolic function and a more sophisticated analysis of the left and right ventricular function using speckle tracking and strain analysis, would have provided more information. However, this was not a feasible study to perform.

## Conclusions

Our study on not preselected patients at a cardiac outpatient office did not reveal any relevant long-term effects on electrocardiographic and echocardiographic parameters three and six months after SARS-CoV2 infection. Selection bias may have limited this study; however, patients at a cardiac outpatient center tend to be sicker than those of a general practitioner. In our study, most participants were presumably infected with the ß-variant of the virus. However, new or other SARS-CoV2 variants may show different cardiotropic properties and different long-term cardiac effects. As a result, more studies are needed to confirm our findings.
